# Iron-biofortification in rice by the introduction of three barley genes participated in mugineic acid biosynthesis with soybean *ferritin* gene

**DOI:** 10.3389/fpls.2013.00132

**Published:** 2013-05-14

**Authors:** Hiroshi Masuda, Takanori Kobayashi, Yasuhiro Ishimaru, Michiko Takahashi, May S. Aung, Hiromi Nakanishi, Satoshi Mori, Naoko K. Nishizawa

**Affiliations:** ^1^Research Institute for Bioresources and Biotechnology, Ishikawa Prefectural UniversityIshikawa, Japan; ^2^Graduate School of Agricultural and Life Sciences, The University of TokyoTokyo, Japan

**Keywords:** anemia, iron, zinc, rice, mugineic acid, biofortification, ferritin, IDS3

## Abstract

Iron deficiency is a serious problem around the world, especially in developing countries. The production of iron-biofortified rice will help ameliorate this problem. Previously, expression of the iron storage protein, ferritin, in rice using an endosperm-specific promoter resulted in a two-fold increase in iron concentration in the resultant transgenic seeds. However, further over expression of ferritin did not produce an additional increase in the seed iron concentration, and symptoms of iron deficiency were noted in the leaves of the transgenic plants. In the present study, we aimed to further increase the iron concentration in rice seeds without increasing the sensitivity to iron deficiency by enhancing the uptake and transport of iron via a ferric iron chelator, mugineic acid. To this end, we introduced the soybean *ferritin* gene (*SoyferH2*) driven by two endosperm-specific promoters, along with the barley nicotianamine synthase gene (*HvNAS1*), two nicotianamine aminotransferase genes (*HvNAAT-A* and -*B*), and a mugineic acid synthase gene (*IDS3*) to enhance mugineic acid production in rice plants. A marker-free vector was utilized as a means of increasing public acceptance. Representative lines were selected from 102 transformants based on the iron concentration in polished seeds and ferritin accumulation in the seeds. These lines were grown in both commercially supplied soil (iron-sufficient conditions) and calcareous soil (iron-deficient conditions). Lines expressing both ferritin and mugineic acid biosynthetic genes showed signs of iron-deficiency tolerance in calcareous soil. The iron concentration in polished T_3_ seeds was increased by 4 and 2.5 times, as compared to that in non-transgenic lines grown in normal and calcareous soil, respectively. These results indicate that the concomitant introduction of the *ferritin* gene and mugineic acid biosynthetic genes effectively increased the seed iron level without causing iron sensitivity under iron-limited conditions.

## Introduction

Iron (Fe) is an essential micronutrient for most organisms, including all plants and animals. Fe deficiency is one of the most prevalent micronutrient deficiencies in the world, affecting an estimated two billion people (Stoltzfus and Dreyfuss, 1998) and causing 0.8 million deaths per year worldwide. Fe deficiency is ranked sixth among the risk factors for death and disability in developing countries (WHO, 2002).

There are three basic ways to solve micronutrient deficiencies: micronutrient supplementation, food fortification, and biofortification. Among these options, biofortification does not require specific processing after harvest or a special infrastructure (Grusak and DellaPenna, 1999; Mayer et al., 2008). Therefore, it is beneficial for people who may find it difficult to change their dietary habits because of financial, cultural, regional, or religious restrictions. Rice is a particularly suitable target for biofortification because Fe-deficiency anemia is a serious problem in developing countries where rice is a major staple crop (Juliano, 1993; WHO, 2002). Rice endosperm accumulates a high concentration of starch and becomes the edible part of the seed after milling, at which point the seeds are known as polished or white seeds (Juliano, 1993). Rice seeds, and especially endosperm, contain low levels of most minerals, including micronutrient metals (Grusak and Cakmak, 2005); therefore, it is important to improve the Fe concentration in polished seeds.

There are many trials to improve mineral nutrition in rice seeds by traditional breeding or transgenic methods. IR68144 was produced using traditional breeding by International Rice Research Institute (IRRI). This variety contains over two times higher Fe concentration in seeds than local varieties in Philippine (Gregorio et al., 2000). IR68144 improved the Fe states of Philippine women better than a local rice variety (Haas et al., 2005).

Nowadays, transgenic approach can be used for the production of micronutrient-fortified rice varieties. The first transgenic approach to increase Fe concentration in rice seeds is the enhancement of Fe accumulation in rice seeds by *ferritin* gene expression under the control of endosperm-specific promoters. Goto et al. (1999) generated transgenic rice plants that expressed the soybean *ferritin* gene, *SoyferH1*, in endosperm using the endosperm-specific, 1.3-kb *OsGluB1* rice promoter; the transformants showed increased Fe accumulation in brown seeds. A few reports have described the production of Fe-biofortified rice through the endosperm-specific expression of ferritin (Lucca et al., 2002; Vasconcelos et al., 2003). Furthermore, Qu et al. (2005) expressed *SoyferH1* under the control of both the *OsGlb* promoter and 1.3-kb *OsGluB1* promoter to further increase the seed Fe concentration. However, increasing the level of ferritin expression in rice seeds did not significantly increase the Fe concentration; moreover, it caused symptoms of iron deficiency in the leaves of the transgenic plants. Thus, the enhancement of ferritin expression may not be sufficient to further increase the Fe concentration in rice grains. Qu et al. (2005) proposed that in addition to increased Fe storage in seeds, enhanced Fe uptake from the soil and enhanced translocation within the plant body are required to further improve the Fe biofortification of rice seeds.

Fe uptake, translocation, and homeostasis in rice are beginning to be understood at the molecular level (Grusak et al., 1999; Bashir et al., 2010). Graminaceous plants synthesize and secrete mugineic acid family phytosiderophores (MAs), which are natural Fe(III) chelators that take up Fe from the rhizosphere (Figure S1; Takagi, 1976; Mihashi and Mori, 1989). Nicotianamine (NA) is biosynthesized from *S*-adenosyl methionine via NAS (Higuchi et al., 1999). In graminaceous plants, including rice, deoxymugineic acid (DMA) is synthesized from NA by NA aminotransferase (NAAT) and DMA synthase (DMAS) (Takahashi et al., 1999; Bashir et al., 2006; Inoue et al., 2009). In barley and other graminaceous plants, other types of MAs are synthesized from DMA by Fe deficiency-specific clone no. 2 (IDS2) and no. 3 (IDS3: also known as mugineic acid synthase) (Nakanishi et al., 2000; Kobayashi et al., 2001). Among graminaceous plants, barley is highly tolerant to Fe deficiency and possesses a series of biosynthetic genes for MAs, including *HvNAS1*, *HvNAAT-A*, *HvNAAT-B*, *HvDMAS1*, *IDS2*, and *IDS3*, which are up-regulated in Fe-deficient barley roots (Higuchi et al., 1999; Takahashi et al., 1999; Nakanishi et al., 2000; Bashir et al., 2006). In contrast, rice lacks *IDS2* and *IDS3* and secretes only DMA. This is thought to be one of the reasons why barley has greater tolerance to Fe deficiency than rice (Kobayashi et al., 2001). In rice, Fe(III)-DMA complexes are thought to be absorbed through the transporter OsYSL15 (Inoue et al., 2009; Lee et al., 2009a). In addition to its function in Fe uptake, Fe(III)-DMA is transported into rice seeds more efficiently, as compared to Fe(III) through the rice plant body (Tsukamoto et al., 2009). Based on our knowledge of the mechanism of Fe uptake and transport by MAs in graminaceous plants, transgenic rice lines with increased tolerance to Fe deficiency were produced. Suzuki et al. (2008) cultivated three types of transgenic rice lines carrying the barley genes responsible for MAs biosynthesis (*HvNAS1*, *HvNAS1*, *HvNAAT-A*, *HvNAAT-B*, and *IDS3*) in a field with calcareous soil. Rice lines expressing *HvNAS1* or *IDS3* showed Fe-deficiency tolerance, possibly because of improved Fe uptake and translocation caused by the enhancement of DMA and MA biosynthesis. In addition to DMA, the introduction of *IDS3* conferred MA secretion in rice (Kobayashi et al., 2001). Because MA have greater Fe(III)-complex stability than DMA at a slightly acidic pH (von Wirén et al., 2000), the production of MA via *IDS3* might be advantageous for Fe translocation in rice. Furthermore, because these transformants contained introduced barley genome fragments, expression of the genes responsible for MAs biosynthesis was regulated by their own promoters. In rice, these promoters induced expression in response to Fe deficiency in roots and leaves (Higuchi et al., 2001; Kobayashi et al., 2001). Thus, these genes are expected to be expressed when and where the requirement for Fe is elevated.

The Fe concentration in seeds of rice lines transformed with *HvNAS1*, *HvNAS1*, *HvNAAT-A*, *HvNAAT-B*, and *IDS3* was analyzed after cultivation in the field in Fe-sufficient (Andosol) or Fe-deficient (calcareous) soil (Masuda et al., 2008; Suzuki et al., 2008). The *IDS3* rice line showed an increased Fe concentration in polished seeds up to 1.25–1.4 times that in non-transgenic (NT) rice following cultivation in Andosol and calcareous soil (Masuda et al., 2008; Suzuki et al., 2008).

In the present report, we produced Fe biofortified rice by the concomitant introduction of soybean *ferritin* gene (*SoyferH2*) under the control of the *OsGluB1* and *OsGlb* promoters and barley genes encoding enzymes for MAs biosynthesis (genome fragments of *HvNAS1*, *HvNAAT-A*, *HvNAAT-B*, and *IDS3*). The transformants exhibited Fe-deficiency tolerance in calcareous soil. The Fe concentration in T_3_ polished seeds was increased 4 and 2.5 times, as compared to that in NT plants grown in commercially supplied soil and calcareous soil, respectively. We found that Fe biofortification through the concomitant introduction of genes encoding ferritin and biosynthetic enzymes for MAs effectively increased the seed Fe level and improved Fe sensitivity under Fe limitation, which is caused in case of single introduction of *ferritin*.

## Materials and methods

### Plant materials

The *Japonica* rice (*Oryza sativa* L.) cultivar Tsukinohikari was used as the NT control and for transformation.

### Vector construction, confirmation of vector construct and rice transformation

pBIMFN (marker-free vector), which was produced by Nishizawa et al. (2006), was used as the backbone of the binary vector for rice transformation. Using this vector, the Fer-NAS-NAAT-IDS3 and Fer rice transformation vectors were constructed according to the scheme shown in Figures S2, S3, respectively. The constructed vectors were verified by PCR, as shown in Figure S4. For Fer-NAS-NAAT-IDS3 vector, Glbp 5′R primer 5′-ACC AGA TAC AAC GGG TCC CTC-3′ and NAAT 5′R primer 5′-GGT ATC GCC ATT CGC CAA GCC AGT-3′ were used to confirm the gene connection of gene cassette Os*Glb promoter*-*SoyferH2* and *HvNAAT-A, -B*. NAAT 3′F primer 5′-GTC ACT CGC TCT ATC TTG GTC ATT G-3′ and NAS 5′R primer 5′-GTT GAG GAT ACA CTA TTG CTC ATG C-3′ were used to confirm the gene connection of *HvNAAT-A, -B* genome and *HvNAS1* genome. NAS 3′F primer 5′-GAC TAA GCG TCG TCA TGA ACC TGT G-3′ and tNos 3′F primer 5′-GAA TCC TGT TGC CGG TCT TGC G-3′ were used to confirm the gene connection of *HvNAS1* genome and Os*GluB1 promoter*-*SoyferH2* gene construct. GluBp 5′R primer 5′-TGA ACA GTC GTG CTC ACG GTC-3′ and IDS3g 5′R primer 5′-AAC ACA GTA TAG ACG CAA GTG TTC A-3′ were used to confirm the gene connection of *OsGluB1 promoter*-*SoyferH2* gene construct and *IDS3* genome. For Fer vector, Glbp 5′R primer and GluBp 5′R primer were used to confirm the gene connection of gene cassette Os*Glb promoter*-*SoyferH2* and *OsGluB1 promoter*-*SoyferH2*. Sequence of PCR product was checked by ABI PRISM 310(ABI) and compared to the expected sequence from the data.

* Agrobacterium tumefaciens* (strain C58) was used to introduce the constructs into *O. sativa* L. cv. Tsukinohikari using the method outlined in Hiei et al. (1994). Transgenic calli were serially selected by 10, 20, and 30 mg/L concentrations of hygromycin. 30 mg/L concentration of hygromycin was also included in regeneration medium and root elongation medium.

### Greenhouse cultivation

T_0_ regenerate plants as well as T_1_ Fer-NAS-NAAT-IDS3 lines, Fer lines, and NT plants were germinated on Murashige and Skoog (MS) medium and cultivated in 3.5 CL pots (1,000-ml volume; Kaneya, Aichi, Japan) containing a 2:1 mixture of Bonsol-ichigou (commercially supplied soil used for rice cultivation in Japanese nurseries; Sumitomo Chemicals, Tokyo, Japan) and vermiculite (Green Tec, Tochigi, Japan). The soil was evenly fertilized with 3.5 g of Long Total-70 and Long Total-140 (slow-release fertilizers; JCAM AGRI Co. Ltd., Tokyo, Japan; N:P:K, 13:11:13 and 2% Mg, 0.1% Mn, 0.06% B, 0.20% Fe, 0.050% Cu, 0.015% Zn, and 0.020% Mo as micronutrients) per plant. The plants were grown in a greenhouse under natural light conditions, with 14 h of light at 30°C and 10 h of dark at 25°C. Six plants each of the T_2_ Fer-NAS-NAAT-IDS3 lines (1-12, 22-4, and 34-11), Fer line 13-6, and NT plants were cultivated in commercially supplied soil and vermiculite as described above under Fe-sufficient conditions. The T_2_ plants were also cultivated in calcareous soil (pH = 8.9) obtained from Takaoka City, Toyama, Japan (Nihonkai Kougyo, Toyama, Japan) in 3.5 CL pots with 3.5 g of Long Total-70 and -140 under Fe-deficient conditions. The seeds obtained from greenhouse cultivation were used for metal concentration analysis.

For Northern blot analysis, T_2_ Fer-NAS-NAAT-IDS3 lines 1-12, 22-4, and 34-11 were germinated on MS medium. After 3 weeks, six seedlings from each line were transferred to nutrient solution [2 mM Ca(NO_3_)_2_, 0.5 mM MgSO_4_, 0.7 mM K_2_ SO_4_, 0.1 mM KCl, 0.1 mM KH_2_ PO_4_, 0.1 mM Fe(III)-EDTA, 10 mM H_3_ BO_3_, 0.5 μM MnSO_4_, 0.5 μM ZnSO_4_, 0.2 μM CuSO_4_, and 0.01 μM (NH_4_)_6_ Mo_7_ O_25_] and grown in a greenhouse under the conditions described above. The pH of the culture solution was adjusted daily to between 5.5 and 5.8 with 1 N HCl. After 8 days, the plants were transplanted to fresh nutrient solution without Fe(III)-EDTA and cultivated for 1 week. Next, the leaf chlorophyll level was measured using a SPAD-502 chlorophyll meter (Konica Minolta, Tokyo, Japan), and leaves and roots were harvested for Northern blot analysis.

### Genomic PCR

For each line, leaf samples were harvested and DNA was extracted using an automated genomic DNA isolation system (NA-2000; Kurabo, Osaka, Japan). Next, genomic PCR was performed using the following primers. The *OsGluB1* promoter *SoyferH2* construct was detected using the *OsGluB1* promoter FW primer (5′-CAG CTC TCC GTG GTC AGA TGT G-3′) and *SoyferH2* RV primer (5′-GCC ACA CAC CAT GAC CCT TTC CAA C-3′). The *OsGlb* promoter *SoyferH2* construct was detected using the *OsGlb* promoter FW primer (5′-CCA ACC GAT CCA TGT CAC CCT CAA GC-3′) and *SoyferH2* RV primer. *IDS3* genome insertion was detected using the *IDS3* FW primer (5′-AAG CTT ACT GGT TGG ACG GTA TTT CA-3′) and *IDS3* RV primer (5′-GGA TCC ACG GGC CAC ATG ATC CA-3′). *HvNAAT-A* genome insertion was detected using the *HvNAAT-A* FW primer (5′-GTG TTG CAT GTC AAA TGA CCG G-3′) and *HvNAAT-A* RV primer (5′-CTA CTC CCT CTG TCC CAA AAT AAC TG-3′). *HvNAAT-B* genome insertion was detected using the *HvNAAT-B* FW primer (5′-CCG AAA ATG CAT CCA ACA TAA TTA C-3′) and *HvNAAT-B* RV primer (5′-GCC AAT GTA ACT TCA CTA ACA TAA C-3′). *HvNAS1* genome insertion was detected using the *HvNAS1* FW primer (5′-CGG TGG AGG TAA TAG CCC TAC GTC-3′) and *HvNAS1* RV primer (5′- GGA GGC AGT CCT GTT GTG GCA TTC-3′).

### Northern blot analysis

The ORFs for *HvNAS1* (AB010086), *HvNAAT-A* (D88273), *HvNAAT-B* (AB005788), and *IDS3* (AB024058) were used to synthesize *HvNAS1*, *HvNAAT-A*, *HvNAAT-B*, and *IDS3* probes using the primers described in Suzuki et al. (2006). This fragment was labeled with [a-^32^P]-dATP using the random labeling method; the labeled DNA was purified using a ProbeQuant G-50 Micro Column (Pharmacia, Uppsala, Sweden). Total RNA from the roots and shoots was extracted using the sodium dodecyl sulfate (SDS)-phenol method. Aliquots of the total RNA (20 μg per lane) were separated on 1.4% (w/v) agarose gels. Blotting, hybridization, and radioactive detection were performed as described previously (Ogo et al., 2006; Suzuki et al., 2006).

### Western blot analysis

Six T_2_ or six T_3_ mature seeds of the Fer-NAS-NAAT-IDS3, Fer, and NT lines were harvested and examined for ferritin expression by Western blotting. The seeds were homogenized with a mortar and pestle, soaked in extraction buffer (4% SDS, 5% 2-mercaptethanol, 20% glycerol, 20 mM Tris-HCl, 8 M urea, and 0.1% bromophenol blue, pH 6.8), and shaken for 30 min. The resulting extracts were centrifuged at 13,000 rpm for 20 min and supernatant fractions were collected. Protein separation by SDS-polyacrylamide gel electrophoresis, transfer to polyvinylidene fluoride membranes, and detection with antibodies were performed as described in Goto et al. (1999).

### Quantitative real-time RT-PCR analysis of *soyferH2* in FE deficient leaves

Plants were germinated on MS medium. After 3 weeks, three plants from each line were cultivated in nutrient solution with Fe(III)-EDTA for 2 weeks and then in nutrient solution without Fe(III)-EDTA for 13 days. Total RNA was extracted from the second newest leaf of each plant by using an RNeasy Plant Mini Kit (Qiagen, Tokyo, Japan). First-strand cDNA was synthesized using ReverTra Ace qPCR RT Master Mix with gDNA remover (TOYOBO, Osaka, Japan). Real-time RT-PCR was carried out using the StepOnePlus™ Real-Time PCR System (Applied Biosystems, Tokyo, Japan) with SYBR Premix Ex Taq II (Takara, Shiga, Japan). *SoyferH2* forward (5′-GCT TTT ATC TCT CGC CCG TTG-3′) and *SoyferH2* reverse (5′-CAT TGT GTG CAA TCG GAA CAG C-3′) primers were used. Transcript levels were normalized to the expression levels of alpha-*Tubulin*, as determined using the primers alpha-*Tubulin* forward (5′-TCT TCC ACC CTG AGC AGC TC-3′) and alpha-*Tubulin* reverse (5′-AAC CTT GGA GAC CAG TGC AG-3′). The sizes of the amplified fragments were confirmed by agarose gel electrophoresis.

### Metal concentration analysis

A seed metal concentration analysis was performed according to the method of Masuda et al. (2008); Masuda et al. (2009). Brown seeds were collected randomly from the ear of the main tiller (the tiller in the center or the largest among all tillers in one plant). Ten seeds from each plant were dried overnight at 80°C in a heat drying machine. After determining the dry weight of each sample, the seeds were digested in 1 ml of 13 M HNO_3_ and 1 ml of 8.8 M H_2_ O_2_ (Wako, Osaka, Japan) at 200°C for 20 min with MARS Xpress (CEM, Matthews, NC, USA). After digestion, the samples were diluted to a volume of 5 ml and analyzed via inductively coupled plasma atomic emission spectrometry (SPS1200VR; Seiko, Tokyo, Japan).

To obtain polished seeds, 30 brown seeds from the ear of the main tiller were placed into a 2-ml tube and shaken vigorously for 150 s at 2500 rpm for four cycles using a Multi-Beads Shocker (Yasui Kikai, Osaka, Japan). Ten well polished seeds from each line were selected and dried overnight, weighed, digested, and then metal concentration was analyzed as described above.

Rice husks (approximately 100 mg) were also dried overnight, weighed, digested, and subjected to metal concentration analysis as described above.

### Statistics

Student's *t*-test was used for each sample set to compare the data for the significant differences. The level of significance was set at *P* < 0.05.

## Results

### Production of fer-NAS-NAAT-IDS3 transgenic lines

**Figure 1:**
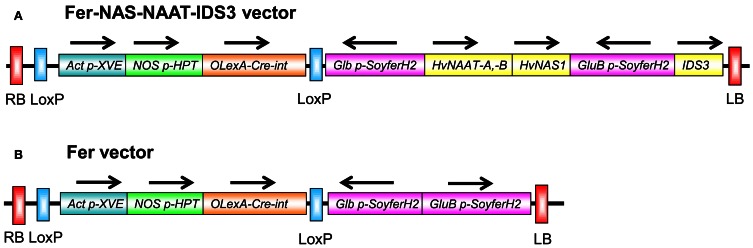
**T-DNA regions of the constructs introduced into rice. (A)** Fer-NAS-NAAT-IDS3 vector. **(B)** Fer vector. Arrows show the direction of transcription. RB, right border; LB, left border; *Act p*, promoter region of rice *OsActin1* (Os03g0718100); *XVE*, estradiol receptor-based transcription factor gene (Zuo et al., 2001); *NOS p*, nopaline synthase (NOS) gene promoter (Zuo et al., 2001); *HPT*, hygromycin phosphotransferase gene (K01193); *OLexA*, eight copies of the *LexA* operator sequence fused to the −46 CaMV *35S* promoter (Zuo et al., 2001); *Cre-int*, the coding sequence of Cre recombinase interrupted by an intron (Zuo et al., 2001); *Glb p*, promoter region of the 26-kDa *OsGlb1* gene (AY427575) (Qu and Takaiwa, 2004); *SoyferH2*, soybean *ferritin* gene (AB062754) (Masuda et al., 2001); *HvNAAT-A,-B*, barley genome fragment containing the nicotianamine aminotransferase genes *HvNAAT-A* and *HvNAAT-B* (AB024006); *HvNAS1*, barley genome fragment containing a nicotianamine synthesis gene (Higuchi et al., 2001); *GluB p*, 2.3-kb promoter region of *OsGluB1* (AY427569) (Qu and Takaiwa, 2004); *IDS3* genome, barley genome fragment containing a mugineic acid synthesis gene (AB024007). Each gene expression cassette, except the *HvNAS1* genome, *HvNAAT-A,-B* genome, and *IDS3* genome fragments, possessed the *A. tumefaciens* NOS gene terminator (AF485783) *Tnos* at the 3′ end of the *ORF*. The T-DNA region between two *LoxP* sites can be removed from the vector by estradiol treatment (Cre/loxP system; Zuo et al., 2001; Figure S8). The vector backbone was derived from pBIMFN (Nishizawa et al., 2006).

To produce transgenic rice lines that concomitantly expressed soybean ferritin and barley enzymes for MAs biosynthesis, the transformation vector Fer-NAS-NAAT-IDS3 was produced (Figure 1A). This vector contained the *OsGluB1* promoter-*SoyferH2*, *OsGlb* promoter-*SoyferH2*, a 5-kb *HvNAS1* genome fragment, an 11-kb *HvNAAT-A,-B* genome fragment, and a 5-kb *IDS3* genome fragment in the marker-free vector pBIMFN (Nishizawa et al., 2006). This vector was used for rice transformation and 102 lines were produced (referred to as Fer-NAS-NAAT-IDS3 lines).

To determine the advantage of introducing biosynthetic genes for MAs together with the *ferritin* gene, another rice transformation vector, Fer vector, which included the *OsGluB1* promoter-*SoyferH2* and *OsGlb* promoter-*SoyferH2* but no genes for MAs biosynthesis, was also produced (Figure 1B). Using this vector, 14 lines were obtained (referred to as Fer lines). The efficiency of transgenic plant production was lower in the Fer-NAS-NAAT-IDS3 lines than in the Fer lines and other transgenic lines of Tsukinohikari (data not shown). Among the transformants obtained, we screened desirable lines on the basis of the Fe concentration level in polished seeds rather than the transgene expression.

*OsGluB1* promoter-*SoyferH2* and *IDS3* genome insertion in the Fer-NAS-NAAT-IDS3 lines was detected by genomic PCR. Among the 102 lines, insertion of the *OsGluB1* promoter-*SoyferH2* and *IDS3* was confirmed in Fer-NAS-NAAT-IDS3 lines 1, 4, 12, 13, 14, 16, 18, 19, 21, 25, 27, 30, and 34 (data not shown). Among the 14 Fer lines, insertion of the *OsGluB1* promoter-*SoyferH2* was confirmed in lines 2, 11, 13, and 14 (data not shown). Next, the transgenic lines were cultivated in a greenhouse to obtain seeds.

### FE concentration measurement and ferritin accumulation in T_1_ seeds

**Figure 2:**
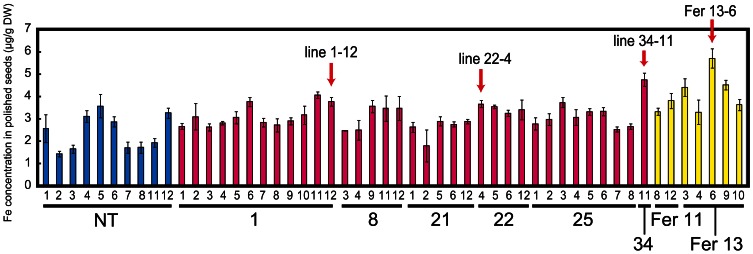
**Fe concentration in polished T_2_ seeds.** NT, non-transgenic line (blue bars); 1, 8, 21, 22, 25, and 34, Fer-NAS-NAAT-IDS3 lines (red bars); Fer 11 and Fer 13, Fer lines (yellow bars). Small numerals under each bar indicate the number of sub-lines. The lines indicated with arrows were selected for further analysis. Bars represent the means ± standard errors of three independent analyses (*n* = 3).

**Figure 3:**
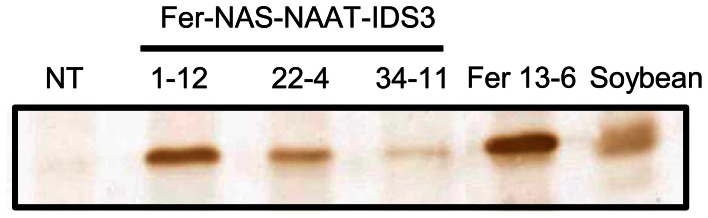
**Ferritin accumulation in T_2_ seeds as determined by Western blot analysis.** NT, non-transgenic seeds; 1-12, 22-4, and 34-11, T_2_ seeds of the Fer-NAS-NAAT-IDS3 lines; Fer 13-6, T_2_ seeds of Fer line 13-6; Soybean, protein extracted from soybean seeds used as a positive control for soybean ferritin.

Among the transformants obtained, we screened desirable lines on the basis of Fe concentration level in polished seeds rather than transgene expression. Therefore, after harvest, the Fe concentration in polished T_1_ seeds was measured. The Fe concentration was around two to three times higher in Fer-NAS-NAAT-IDS3 lines 8, 14, 21, 22, 25, and 34, as compared to that in the NT line (Figure S5). These Fer-NAS-NAAT-IDS3 lines and Fer lines 11 and 13 were cultivated in a greenhouse and the Fe concentration in polished T_2_ seeds was analyzed (Figure 2). Among the Fer-NAS-NAAT-IDS3 lines, 1-12, 22-4, and 34-11 showed especially high Fe concentrations. These lines also exhibited normal yields (data not shown) and were therefore selected for further cultivation and detailed analysis. Among the Fer lines, 13-6 had a higher Fe concentration than the other Fer sub-lines. This line was also selected for further analysis. SoyferH2 accumulation in brown seeds of the selected lines (Fer-NAS-NAAT-IDS3 lines 1-12, 22-4, and 34-11, and Fer line 13-6) was confirmed (Figure 3).

### Hydroponic culture under FE-deficient conditions and gene expression patterns of *HvNAS1*, *HvNAAT-A*, *HvNAAT-B*, and *IDS3*

**Figure 4:**
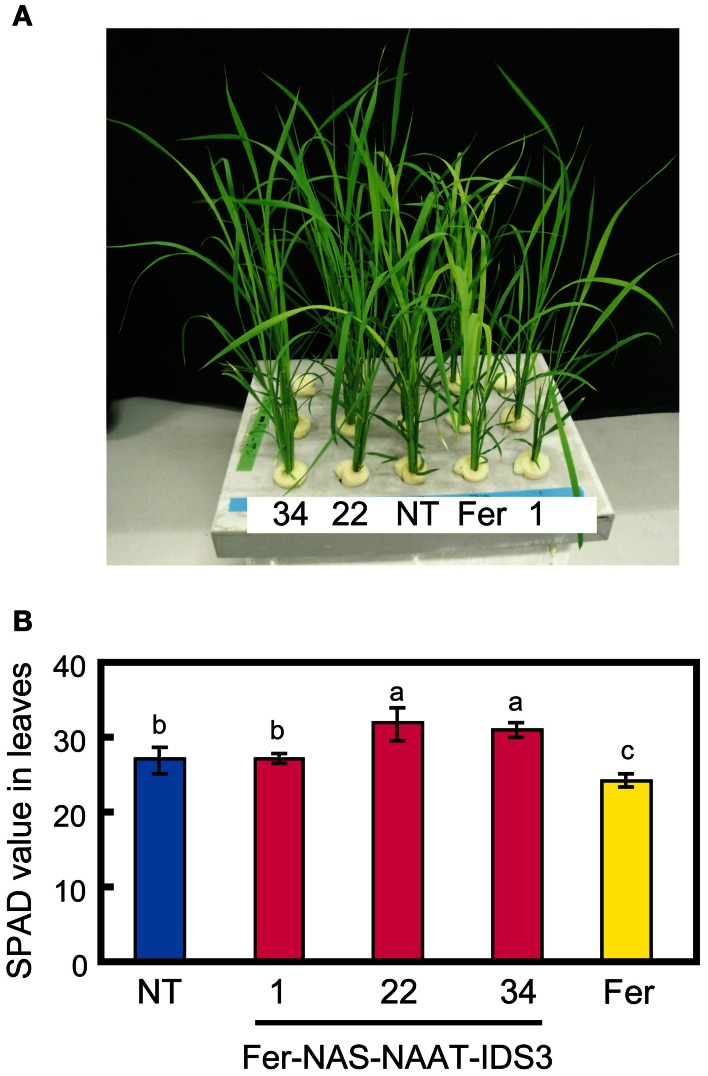
**Fe-deficiency symptoms in hydroponically grown transgenic lines. (A)** Appearance of T_2_ plants cultivated for 7 days in hydroponic culture solution without Fe. **(B)** The SPAD value of T_2_ leaves under Fe-deficient conditions. NT, non-transgenic line; 1, 22, and 34, Fer-NAS-NAAT-IDS3 T_2_ lines 1-12, 22-4, and 34-11, respectively; Fer, Fer line 13-6. Bars represent the means ± standard errors of six independent plants (*n* = 6). Different letters above the bars indicate significant differences (*P* < 0.05) by Student's *t*-test for each line.

To confirm the expression of *HvNAS1*, *HvNAAT-A*, *HvNAAT-B*, and *IDS3* in the Fer-NAS-NAAT-IDS3 lines, plants were grown in hydroponic culture under both Fe-sufficient and -deficient conditions. After 1 week of Fe-deficient cultivation, the leaf color in Fer-NAS-NAAT-IDS3 lines 22-4 and 34-11 remained greener than that in the NT line (Figure 4A), as confirmed by the higher SPAD value (leaf chlorophyll index; Figure 4B). In contrast, the leaves of Fer line 13-6 were yellow and the SPAD value was lower than in the NT line and Fer-NAS-NAAT-IDS3 lines (Figures 4A,B).

**Figure 5:**
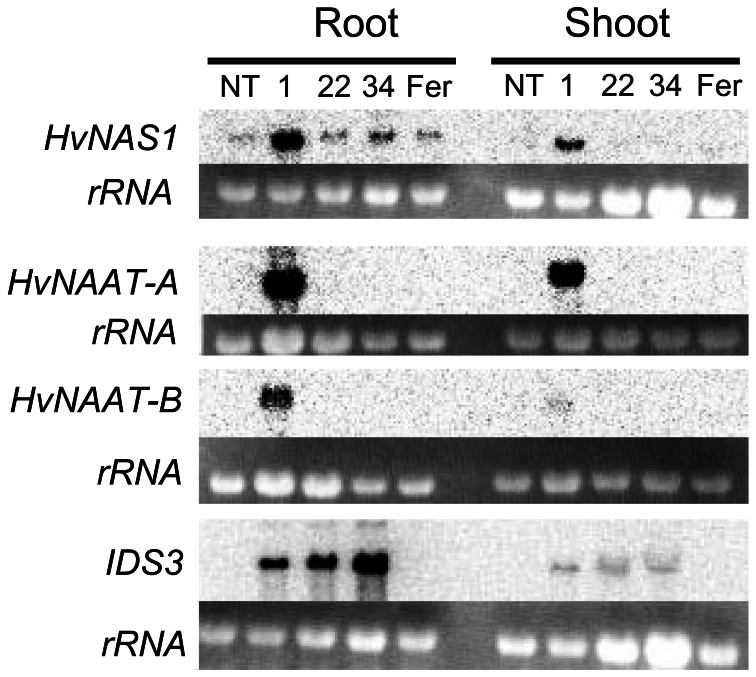
**Expression analysis of mugineic acid synthetic genes by Northern blotting.** Total RNA was extracted from the roots and shoots of T_2_ plants grown for 8 days under Fe-deficient hydroponic culture. NT, non-transgenic line; 1, Fer-NAS-NAAT-IDS3 line 1-12; 22, Fer-NAS-NAAT-IDS3 line 22-4; 34, Fer-NAS-NAAT-IDS3 line 34-11; Fer, Fer line 13-6; rRNA, rRNA band detected by ethidium bromide staining as a loading control.

After Fe-deficiency treatment, total RNA was extracted from the roots and shoots, and the expression of biosynthetic genes for MAs (*HvNAS1*, *HvNAAT-A*, *HvNAAT-B*, and *IDS3*) was analyzed by Northern blot analysis (Figure 5). In Fer-NAS-NAAT-IDS3 line 1-12, all of the introduced genes were expressed strongly in the roots, and they were also expressed in the shoots. In Fer-NAS-NAAT-IDS3 lines 22-4 and 34-11, *IDS3* expression was detected as same as line 1-12. On the other hand, *HvNAAT-A* and *-B* expression was not detected in lines 22-4 and 34-11. In these lines, weak bands were detected by *HvNAS1* hybridization, but the band strength was similar to that in the NT line and Fer line 13-6, in which *HvNAS1* was not introduced. These weak bands might be derived from endogenous *OsNAS1* expression, which could be hybridized to the *HvNAS1* probe because of the strong similarity between *HvNAS1* and *OsNAS1* (84% identity). To confirm the reason for the lack of strong *HvNAS1*, *HvNAAT-A*, and *HvNAAT-B* expression in lines 22-4 and 34-11, insertion of these genes was verified by genomic PCR (Figure S6). The insertion of all of these genes was detected in Fer-NAS-NAAT-IDS3 line 1, but not in lines 22-4 and 34-11, or in the NT line and Fer line 13-6.

### Growth in calcareous soil

**Figure 6:**
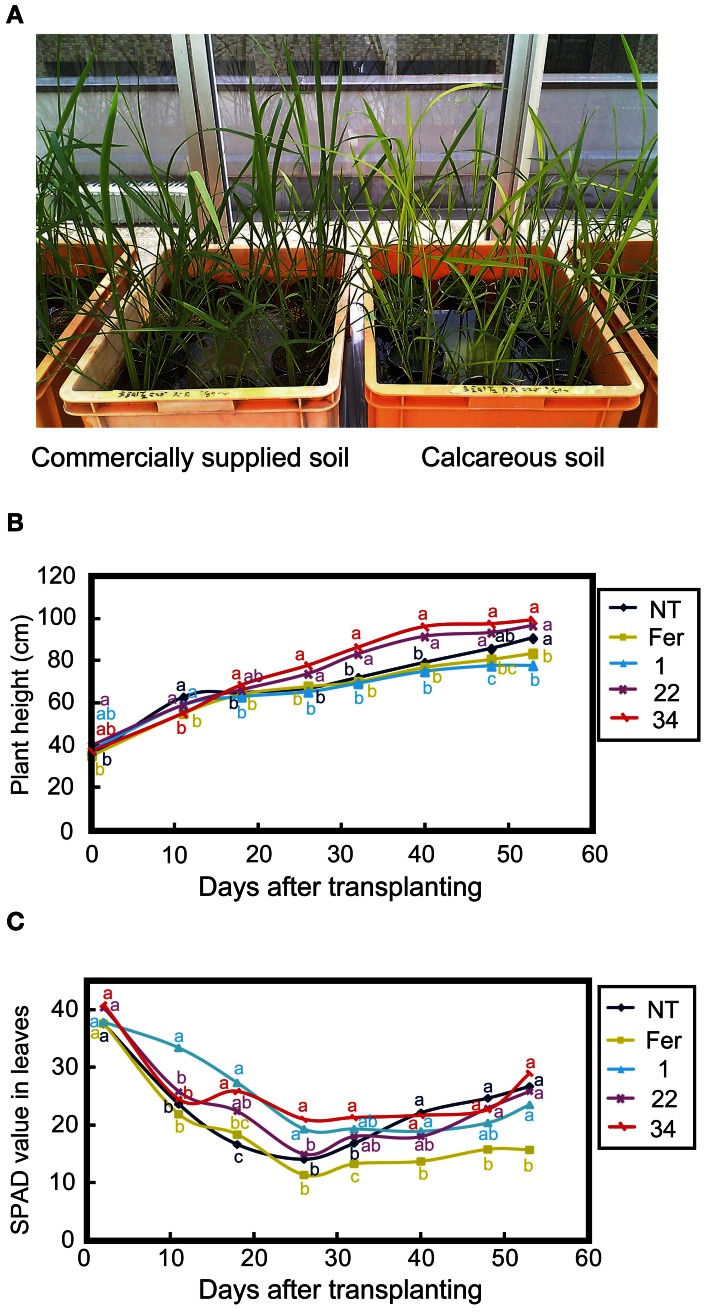
**Calcareous soil cultivation of T_2_ transgenic lines. (A)** Appearance of plants grown in commercially supplied soil and calcareous soil (47 days after germination and 11 days after transplanting to soil). **(B)** Shoot height during calcareous soil cultivation. **(C)** SPAD value of leaves during calcareous soil cultivation. NT, non-transgenic line; Fer, Fer line 13-6; 1, 22, and 34, Fer-NAS-NAAT-IDS3 lines 1-12, 22-4, and 34-11, respectively. The data shown are the average of six plants (*n* = 6). Different letters beside the graph of each line indicate significant differences (*P* < 0.05) by Student's *t*-test for each line.

Suzuki et al. (2008) showed that rice lines with introduced barley *HvNAS1* or the *IDS3* genome fragment grew better than NT plants in calcareous soil. Therefore, we expected that Fer-NAS-NAAT-IDS3 rice would also exhibit Fe-deficiency tolerance, which might be distinguishable in calcareous soil. Therefore, NT plants and T_2_ plants of Fer-NAS-NAAT-IDS3 lines 1-12, 22-4, 34-11 and Fer line 13-6 were grown in both commercially supplied soil (Fe-sufficient conditions) and calcareous soil (Fe-deficient conditions) in a greenhouse (Figure 6A). The shoot height was higher in Fer-NAS-NAAT-IDS3 lines 22-4 and 34-11 than in the NT line and Fer line 13-6 at 30 days after transplanting (DAT) in calcareous soil (Figure 6B). At around 60 DAT, the shoots of Fer-NAS-NAAT-IDS3 lines 22-4 and 34-11 were 15-20 cm higher than those of the NT line and Fer line 13-6. The SPAD values were also higher in Fer-NAS-NAAT-IDS3 lines 1-12, 22-4, and 34-11 than in the NT line and Fer line 13-6 at around 10-30 DAT. The SPAD value was lowest in Fer line 13-6 among all of the lines after 26 DAT (Figure 6C).

### Ferritin accumulation and FE concentration in T_3_ seeds

**Figure 7:**
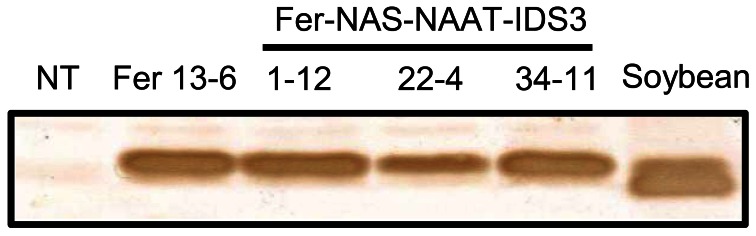
**Ferritin accumulation in T_3_ seeds.** Total protein was extracted from the seeds of six independent T_3_ plants for each line cultivated in calcareous soil, and ferritin was detected by Western blot analysis. NT, non-transgenic seeds; 1-12, 22-4, and 34-11, T_3_ seeds of the corresponding Fer-NAS-NAAT-IDS3 lines; Fer 13-6, T_3_ seeds of Fer line 13-6; Soybean, protein extracted from soybean seeds as a positive control for soybean ferritin.

**Figure 8:**
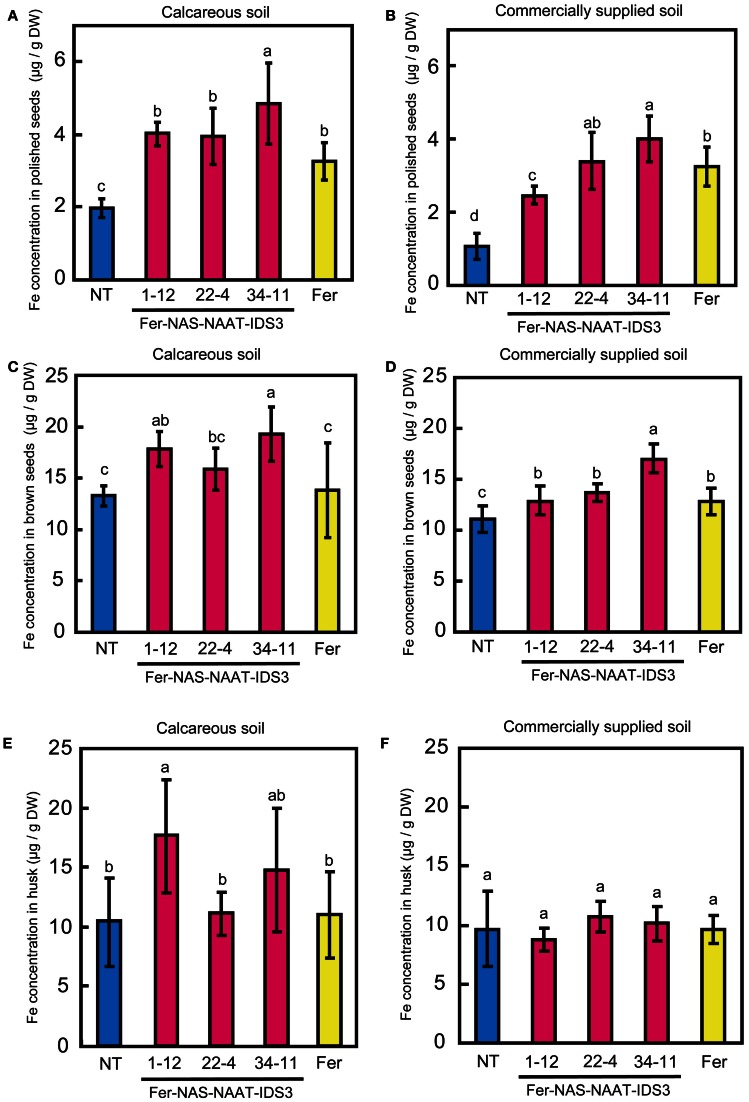
**Fe concentration in T_3_ seeds. (A,B)** Polished seeds. **(C,D)** Brown seeds. **(E,F)** Husk. Plants were cultivated in calcareous soil **(A**, **C**, and **E)** or commercially supplied soil **(B**, **D**, and **F)**. Bars represent the means ± standard errors of six independent plants (*n* = 6). NT, non-transgenic line; 1-12, 22-4, and 34-11, Fer-NAS-NAAT-IDS3 T_3_ lines; Fer, Fer T_3_ line 13-6. Different letters above the bars indicate significant differences (*P* < 0.05) by Student's *t*-test for each line.

**Figure 9:**
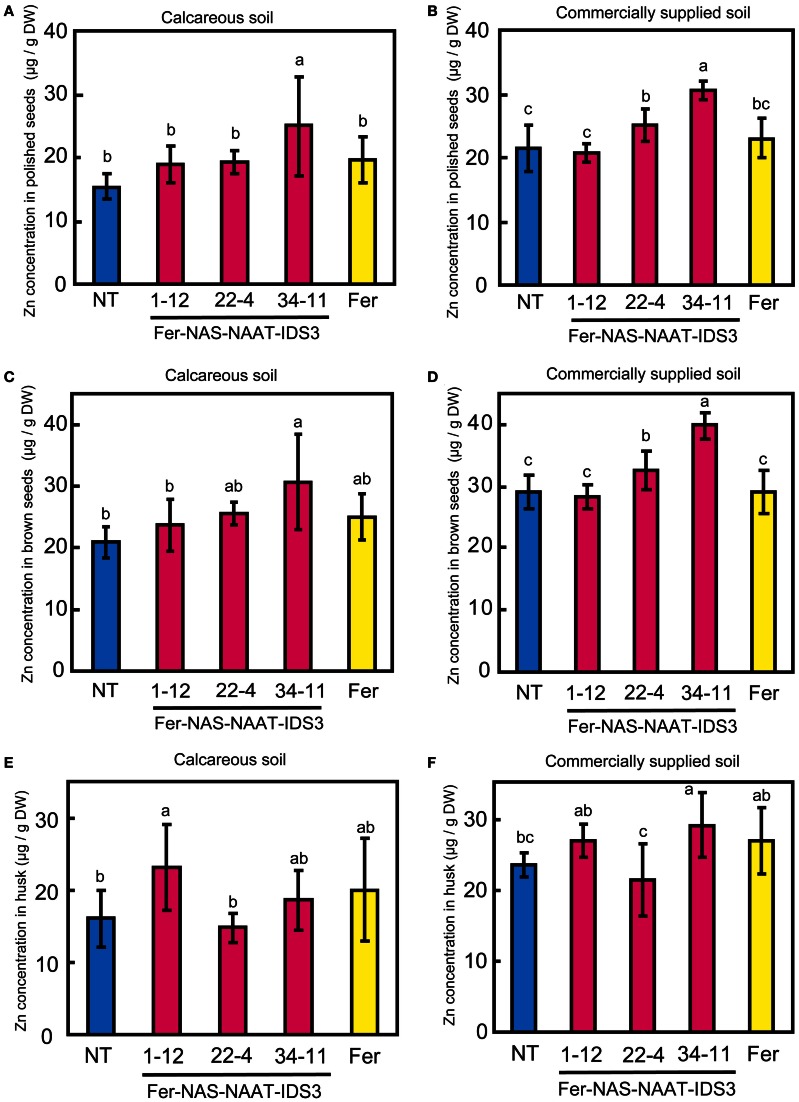
**Zn concentration in T_3_ seeds. (A,B)** Polished seeds. **(C,D)** Brown seeds. **(E,F)** Husk. Plants were cultivated in calcareous soil **(A**, **C**, and **E)** or commercially supplied soil **(B**, **D**, and **F)**. Bars represent the means ± standard errors of six independent plants (*n* = 6). NT, non-transgenic line; 1-12, 22-4, and 34-11, Fer-NAS-NAAT-IDS3 T_3_ lines; Fer, Fer T_3_ line 11-6. Different letters above the bars indicate significant differences (*P* < 0.05) by Student's *t*-test for each line.

Ferritin accumulation was observed in T_3_ seeds of Fer-NAS-NAAT-IDS3 lines 1-12, 22-4, and 34-11, and in Fer line 13-6 seeds by Western blot analysis (Figure 7). Next, the metal concentrations in T_3_ seeds were analyzed (Figures 8, 9). After calcareous soil cultivation, the average Fe concentrations in Fer-NAS-NAAT-IDS3 lines 1-12, 22-4, and 34-11, and in Fer line 13-6 were 4.0, 4.0, 4.9, and 3.3 μg/g, respectively, which is higher than that in the NT line (2.0 μg/g) (Figure 8A). After commercially supplied soil cultivation, the average Fe concentrations in Fer-NAS-NAAT-IDS3 lines 1-12, 22-4, and 34-11, and in Fer line 13-6 were 2.5, 3.4, 4.0, and 3.3 μg/g, respectively, which is also higher than that in the NT line (1.1 μg/g) (Figure 8B). The Fe concentrations in brown seeds of Fer-NAS-NAAT-IDS3 lines 1-12, 22-4, and 34-11 were also higher than that in the NT line following cultivation in either calcareous soil or commercially supplied soil (Figures 8C,D). There was no difference in the Fe concentration in brown seeds between the Fer and NT lines. The Zn concentrations in polished and brown seeds were up to 30% higher in Fer-NAS-NAAT-IDS3 lines 1-12, 22-4, and 34-11, as compared to the NT and Fer lines following cultivation in calcareous soil or commercially supplied soil (Figures 9A–D). There was no remarkable difference in the husk Fe or Zn concentration between the transgenic and NT lines (Figures 8E,F and 9E,F).

In addition to Fe and Zn concentrations, the Fe and Zn contents in endosperm per seed were also higher in Fer-NAS-NAAT-IDS3 lines than in Fer or NT lines following cultivation in both calcareous and commercially supplied soil (Figure S7). Fe and Zn content in bran also tended to increase in Fer-NAS-NAAT-IDS3 lines, with line 34-11 showing significant increase compared to NT lines.

## Discussion

### Generation of Fer-NAS-NAAT-IDS3 vector

To generate new Fe-fortified rice with improved growth under conditions of low Fe availability, we produced transgenic rice lines in which the soybean *ferritin* gene and barley genes responsible for MAs biosynthesis were introduced (Figure 1A). We designed the expression vector Fer-NAS-NAAT-IDS3 with the following issues in mind.

Soybean seeds possess two types of ferritin proteins: SoyferH1 (26.5 kDa) and SoyferH2 (28 kDa) (Masuda et al., 2001). *SoyferH1* can be digested by proteases, which may alter its structure, allowing Fe release, while *SoyferH2* is more resistant to protease digestion than *SoyferH1* (Masuda et al., 2001). Thus, we assumed that the stable ferritin, *SoyferH2*, would be more suitable for Fe accumulation in rice seeds. Therefore, we used *SoyferH2* instead of *SoyferH1* for expression in rice endosperm, which is similar to our previous report (Masuda et al., 2012). In fact, two ferritin bands appeared in soybean seed protein by Western blot analysis, among which the upper band (28 kDa) matched the ferritin bands in our Fer-NAS-NAAT-IDS3 and Fer lines (Figures 3, 7).

Qu et al. (2005) reported that transgenic seeds with introduced *ferritin* under the control of both the 1.3-kb *OsGluB1* promoter and *OsGlb* promoter showed increased accumulation of ferritin compared to those with *ferritin* expressed under the control of either the 1.3-kb *OsGluB1* promoter or the *OsGlb* promoter. In the present study, *SoyferH2* was expressed under the control of both the 2.3-kb *OsGluB1* promoter and the *OsGlb* promoter (Figure 1) in order to achieve high ferritin accumulation in seeds, which is similar to our previous report (Masuda et al., 2012).

For the expression of genes for MAs biosynthesis in rice, the introduction of barley genomic fragments containing the corresponding genes and their promoters has been proven to be highly effective (Higuchi et al., 2001; Kobayashi et al., 2001). However, the introduction of multiple genomic fragments requires a large T-DNA insert, which causes difficulty in transformation. To solve this problem, pBIGRZ1 was used as a suitable binary vector because it allows the introduction of large insertions into the rice genome (Kawasaki, 2003). Using this vector as a backbone, pBIMFN (marker-free vector) was produced (Nishizawa et al., 2006). This vector, which utilizes the Cre/loxP system, allows unnecessary portions of the T-DNA to be removed at any time from Fer-NAS-NAAT-IDS3 lines through estradiol treatment (Zuo et al., 2001) (Figure S8). Usuda et al. (2009) produced transgenic rice lines using this marker-free vector, and successfully removed the marker region from selected transgenic rice lines through estradiol treatment of the seeds. After removal of the two *loxP* regions from Fer-NAS-NAAT-IDS3 lines, all of the remaining transgenes would be derived from rice, soybean, and barley. This should make crops produced using this method easier for the public to accept, as compared to those created using the original binary vector, which includes bacterial selection marker genes such as that encoding hygromycin phosphotransferase or neomycin phosphotransferase II.

### Confirmation of inserted genes

Expression of all transgenes, including *SoyferH2*, *HvNAS1*, *HvNAAT-A*, *HvNAAT-B*, and *IDS3*, was observed only in line 1-12 (Figures 3, 5, and 7). Lines 22-4 and 34-11 did not possess the *HvNAS1*, *HvNAAT-A*, and* HvNAAT-B* transgenes, although *IDS3* expression and ferritin accumulation were observed (Figure S6). In the present study, the Fer-NAS-NAAT-IDS3 construct introduced into rice was large: about 35 kb between the right and left borders (Figure S8). Nakano et al. (2005) introduced a 92-kb wheat genome fragment into rice by *Agrobacterium*-mediated transformation, but none of the four transgenic rice lines possessed the entire sequence; instead, fragments had been inserted. In addition to the 5′ or 3′ sides of the transgene that were missing, the central parts of the transgenes were also found to be missing. Similarly, the insertion of fragments is thought to have occurred in our Fer-NAS-NAAT-IDS3 transgenic lines. The *IDS3* transcripts and ferritin proteins expressed in lines 1-12, 22-4, and 34-11 were similar in size, suggesting a lack of deletion in the expression cassettes for these transgenes (Figures 3, 5, and 7). Lines 22-4 and 34-11 may have lost the central part of the Fer-NAS-NAAT-IDS3 vector, between the *HvNAS1* genome fragment and *HvNAAT-A,-B* genome fragment. Nevertheless, these lines, as well as line 1-12, showed effective Fe accumulation in polished seeds both under Fe-sufficient and -deficient conditions (Figures 8A,B), along with improved tolerance to Fe deficiency (Figure 6), providing promising candidates for future applications as Fe-fortified crops that can tolerate Fe-limiting environments.

### The single introduction of *ferritin* caused sensitivity to FE deficiency but could be overcome by the concomitant introduction of biosynthetic genes for MAS

Under both hydroponic culture and calcareous soil cultivation, Fer-NAS-NAAT-IDS3 lines 1-12, 22-4, and 34-11 showed Fe-deficiency tolerance (Figures 4, 6). Interestingly, Fer line 13 showed the opposite phenotype: sensitivity to Fe deficiency (Figures 4, 6). Wuytswinkel et al. (1999) reported that the overexpression of *ferritin* in tobacco caused abnormal Fe localization and symptoms of Fe deficiency. In our transgenic lines, *SoyferH2* were expressed in Fe-deficient leaves of FC and Fer-NAS-NAAT-IDS3 lines (Figure S9). Data produced using a 44K rice microarray also showed that *OsGluB1* (Os02g0249900) and *OsGlb* (Os05g0499100) were expressed weakly in the leaves of plants grown under Fe-sufficient and -deficient conditions (data not shown). Thus, ectopic *ferritin* expression in the leaves of the Fer line under conditions of Fe deficiency might cause the accumulation and sequestration of Fe, which is needed for growth, leading to an Fe deficiency-sensitive phenotype. This adverse effect was successfully complemented by the concomitant introduction of biosynthetic genes for MAs, as shown in the Fer-NAS-NAAT-IDS3 lines and also in previous reports. Lee et al. (2009b) reported that rice lines with enhanced *OsNAS3* expression showed Fe-deficiency tolerance in addition to an increased Fe concentration in seeds. The overexpression of *NAS* genes together with *ferritin* may also help to avoid Fe deficiency sensitivity caused by introduction of the *ferritin* gene.

In the Fer-NAS-NAAT-IDS3 lines, introduction of the *IDS3* genome fragment is thought to be responsible for the avoidance of sensitivity and further tolerance to Fe deficiency, because lines 22-4 and 34-11 expressed only *IDS3* among the biosynthetic genes for MAs introduced. Suzuki et al. (2008) reported that introduction of the *IDS3* genome fragment into rice conferred Fe-deficiency tolerance in field cultivation. This effect may be attributed to both enhanced production of MAs (DMA plus MA) and increased stability of Fe(III)-MA, as compared to Fe(III)-DMA under some conditions (von Wirén et al., 2000; Kobayashi et al., 2001).

### The combined introduction of *ferritin* and *IDS3* enables effective FE and ZN accumulation in seeds

In Fer-NAS-NAAT-IDS3 lines 22-4 and 34-11, even though the expression of *HvNAS1*, *HvNAAT-A*, and *HvNAAT-B* could not be detected, the Fe concentration in the seeds was the same or higher than that in line 1-12, which expressed all of the introduced genes for MAs biosynthesis (*HvNAS1*, *HvNAAT-A*, *HvNAAT-B*, and *IDS3*) (Figures 5 and 8A,B). In previous field experiments, transgenic rice lines with introduced *HvNAS1* or *HvNAS1* plus *HvNAAT-A* and *HvNAAT-B* did not significantly increase the Fe concentration in seeds (Masuda et al., 2008; Suzuki et al., 2008). On the other hand, rice lines with the introduced* IDS3* genome fragment showed an increased Fe concentration in polished seeds up to 1.25–1.4 times, as compared to that in the NT line in both Andosol soil, which has a normal pH, and calcareous soil (Masuda et al., 2008; Suzuki et al., 2008). Therefore, *IDS3* is thought to be one of the most effective biosynthetic genes for MAs for the Fe biofortification of rice seeds. Rice possesses three *HvNAS1* homologs (*OsNAS1-3*) and six *HvNAAT-A* and *HvNAAT-B* homologs (*OsNAAT1-6*) (Inoue et al., 2003, 2008). In contrast, rice lacks *IDS3* homologs, which synthesize MA from DMA (Nakanishi et al., 2000; Kobayashi et al., 2001). The introduction of* IDS3* to rice confers the ability to produce MA (Kobayashi et al., 2001), which could be advantageous for efficient Fe translocation within plants and might result in increased Fe accumulation in seeds, in addition to Fe-deficiency tolerance. Thus, the increased Fe concentration detected in Fer-NAS-NAAT-IDS3 lines 22-4 and 34-11, as compared to line 1–12, might have been caused by differences in the expression level or pattern of the *IDS3* transgene (Figure 5).

The Fe concentration in brown seeds was increased by up to 30% in the Fer-NAS-NAAT-IDS3 lines but not in the Fer lines, as compared to the NT line under both calcareous soil and commercially supplied soil cultivation (Figures 8C,D). Because *ferritin* was expressed predominantly in the endosperm under the control of the *OsGlb* and *OsGluB1* promoters, the contribution of *ferritin* expression to Fe accumulation is thought to be more obvious in polished seeds than in brown seeds. The concomitant introduction of *IDS3* is thought to have contributed to Fe accumulation in brown seeds by enhancing Fe translocation.

The seed Fe concentration was higher in NT plants grown in calcareous soil, as compared to NT plants grown in commercially supplied soil (Figure 8). This may be because the yield in calcareous soil was low (data not shown) and Fe accumulated well in a limited number of seeds.

The seeds of the Fer-NAS-NAAT-IDS3 lines accumulated both Fe and Zn. In contrast, Fer line 13-6 did not contain higher levels of Zn in its seeds, as compared to the NT line (Figure 9). Masuda et al. (2008) showed that the insertion of *IDS3* into the rice genome increased the Zn concentration by 35% in polished seeds and by 29% in brown seeds in an Andosol field experiment. Suzuki et al. (2008) also showed that the Zn concentration was increased by 37% in brown seeds in a calcareous soil field experiment. Therefore, the increased Zn concentration in the Fer-NAS-NAAT-IDS3 lines may have been caused by the introduction of *IDS3*.

Traditional breeding has also produced high Fe rice such as IR68144. Therefore, for further improvement of Fe concentration in seeds, it will be more efficient to generate higher Fe biofortified rice by transgenic method using a high Fe variety which has already been produced by traditional breeding.

In conclusion, transgenic rice expressing both *ferritin* and the barley MA synthase gene *IDS3* showed increased Fe concentration when the plants were cultivated in both commercially supplied soil and calcareous soil. Fe-deficiency tolerance was also noted under calcareous soil cultivation. These results indicate that the concomitant introduction of *ferritin* and *IDS3* is an effective way to biofortify seeds with Fe without causing Fe-deficiency symptoms under Fe-limited conditions. This method will be especially advantageous for use in Fe-limited environments, including those with a high soil pH and upland cultivation.

### Conflict of interest statement

The authors declare that the research was conducted in the absence of any commercial or financial relationships that could be construed as a potential conflict of interest.
