# Supplementary material for: Iron-biofortification in rice by the introduction of three barley genes participated in mugineic acid biosynthesis with soybean ferritin gene
Source: Front Plant Sci. 2013 May 14;4:132. doi: 10.3389/fpls.2013.00132 (PMC3653162; doi:10.3389/fpls.2013.00132)
Supplement: Supplementary file 1 [file DataSheet1.PDF]

Supplementary Figures of

Iron-biofortification in rice by the introduction of three barley genes participated in mugineic acid biosynthesis with soybean *ferritin* gene

Hiroshi Masuda[1,2], Takanori Kobayashi[1,2], Yasuhiro Ishimaru[2], Michiko Takahashi[2], May Sann Aung[2], Hiromi Nakanishi[2], Satoshi Mori[2] and Naoko K. Nishizawa[1,2]

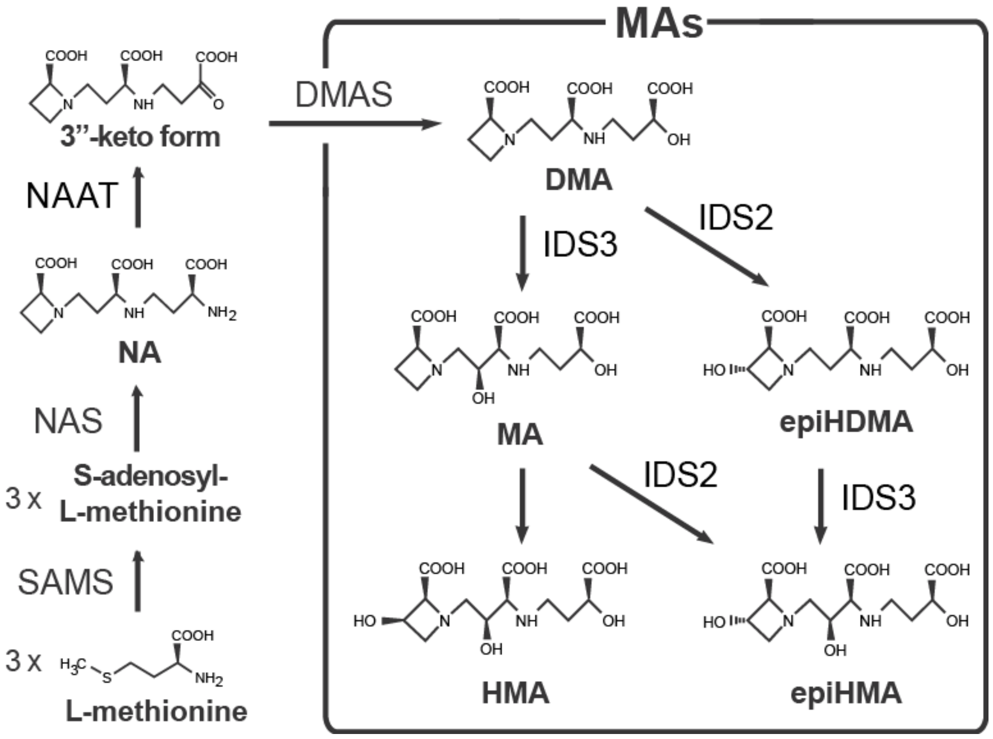

Supplementary Figure 1 | Biosynthetic pathway of mugineic acid family phytosiderophores.

NA, nicotianamine; DMA, 2'-deoxymugineic acid; MA, mugineic acid; HMA, 3-hydroxymugineic acid; epiHDMA, 3-epihydroxy-2'-deoxymugineic acid; epiHMA, 3-epihydroxymugineic acid; SAMS, S-adenosyl-L-methionine synthetase; NAS, NA synthase; NAAT, NA aminotransferase; DMAS, DMA synthase; IDS2, dioxygenase catalyzing the hydroxylation of DMA and MA at position 3; IDS3, MA synthase (dioxygenase catalyzing the hydroxylation of DMA and epiHDMA at the 2' position).

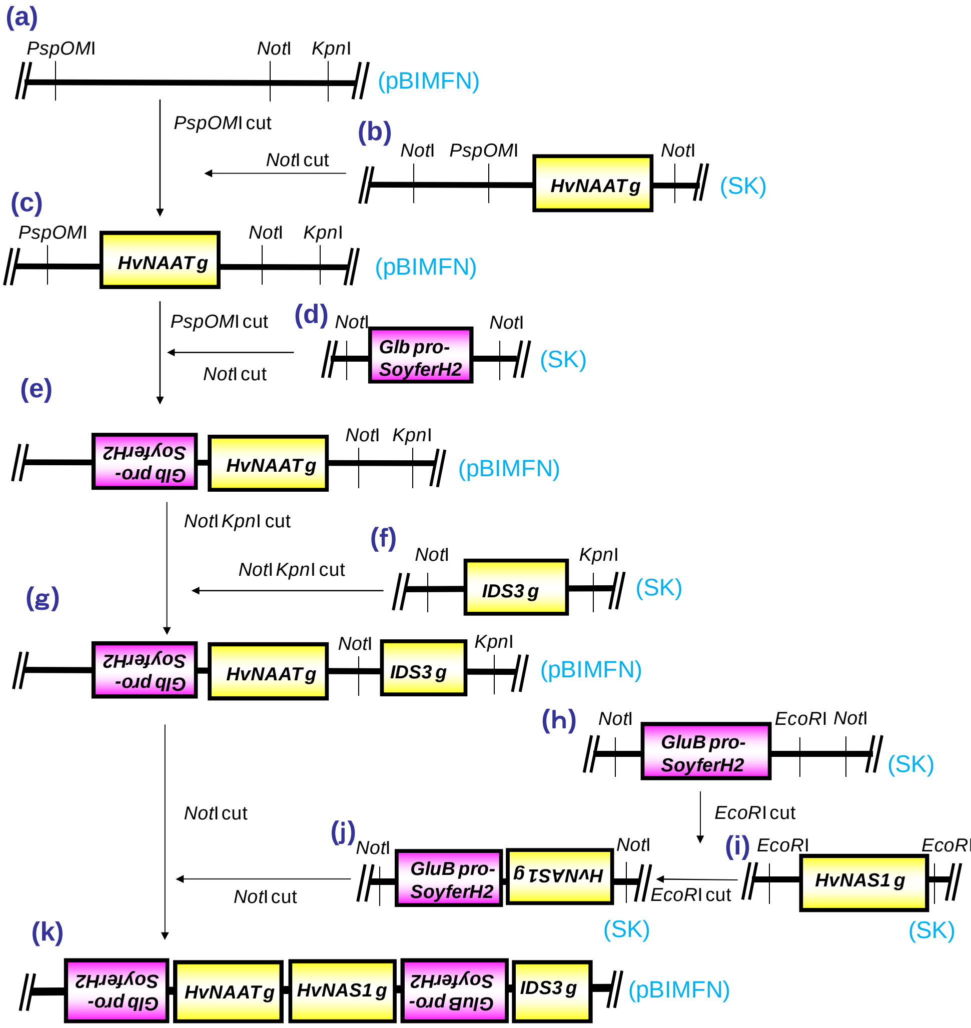

Supplementary Figure 2 | Construction of the Fer-NAS-NAAT-IDS3 transformation vector.

Each vector was excised by the enzymes indicated on or to the side of the arrows, and the two vectors connected by arrows were ligated. The backbone vectors are shown in parentheses. SK, pBluescript SK vector (Stratagene, La Jolla, CA, USA); Vector (**a**), marker-free vector for rice transformation (Nishizawa et al., 2006). Vector (**b**) originated from Takahashi et al. (1999). Vectors (**d**) and (**h**) were generously provided by Dr. F. Takawaiwa (NIAS, Tsukuba, Japan) and Dr. T. Yoshihara (CRIEPI, Abiko, Japan) (Qu et al., 2004, 2005). Vector (**f**) originated from Kobayashi et al. (2001). Vector (**i**) originated from Higuchi et al. (2001). Digestion with *PspOM*I and *Not*I produces ends that are identical and that can be ligated, producing a sequence that can no longer be digested by either *PspOM*I or *Not*I.

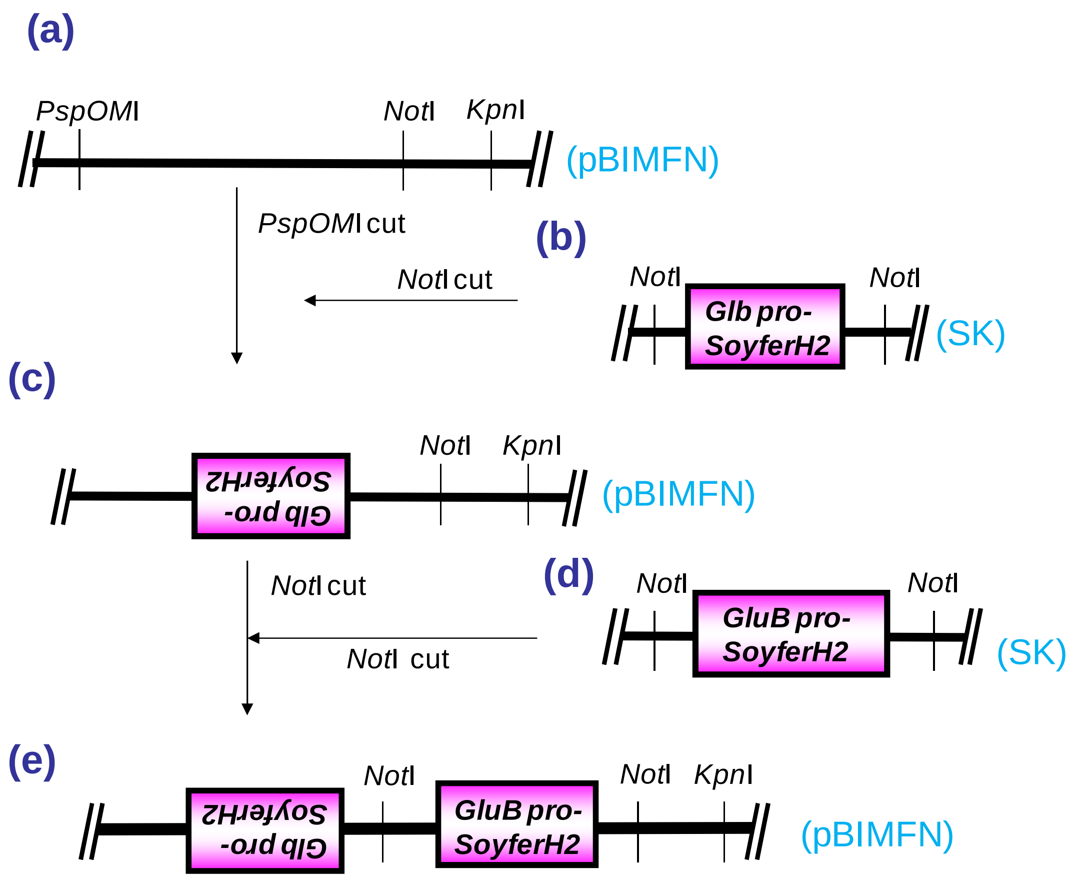

Supplementary Figure 3 | Construction of the Fer transformation vector.

Each vector was excised by the enzymes indicated on or to the side of the arrows, and the two vectors connected by arrows were ligated. The backbone vectors are shown in parentheses. SK, pBluescript SK vector (Stratagene, La Jolla, CA, U.S.A.); Vector (**a**), marker-free vector for rice transformation (Nishizawa et al., 2006). Vectors (**b**) and (**d**) are the same as vectors (**d**) and (**h**) in **Supplementary Figure 2**, respectively. Digestion with *PspOM*I and *Not*I produces ends that are identical and that can be ligated, producing a sequence that can no longer be digested by either *PspOM*I or *Not*I.

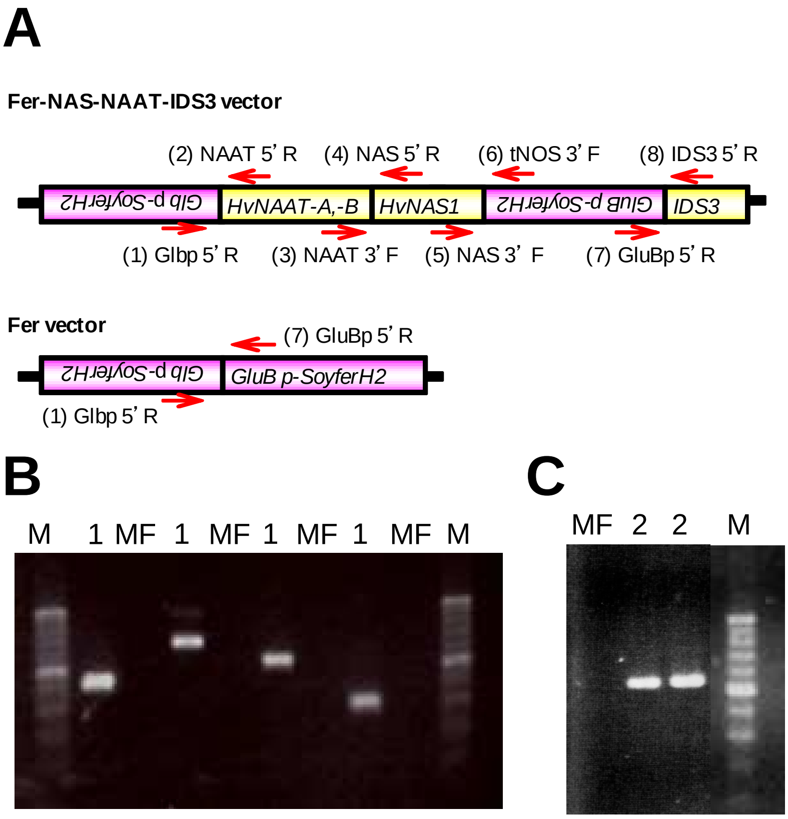

Supplementary Figure 4 | Verification of the constructs by PCR.

(A) Location of the primers used to verify the vector constructs. Arrows show the direction of primers. **(B)** Confirmation of the Fer-NAS-NAAT-IDS3 vector construct. **(C)** Confirmation of the Fer vector construct. M, 100-base pair marker; 1, Fer-NAS-NAAT-IDS3 vector; 2, Fer vector; MF, pBIMFN marker-free vector used as a negative control.

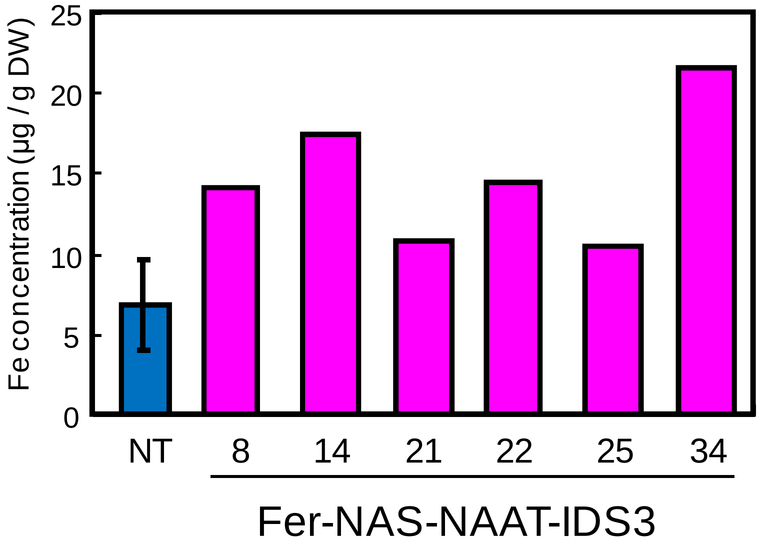

Supplementary Figure 5 | Fe concentration in T_1 polished seeds.

NT, polished seeds of the non-transgenic line (n = 3); Numbers, T_1 polished seeds of the Fer-NAS-NAAT-IDS3 lines (n = 1).

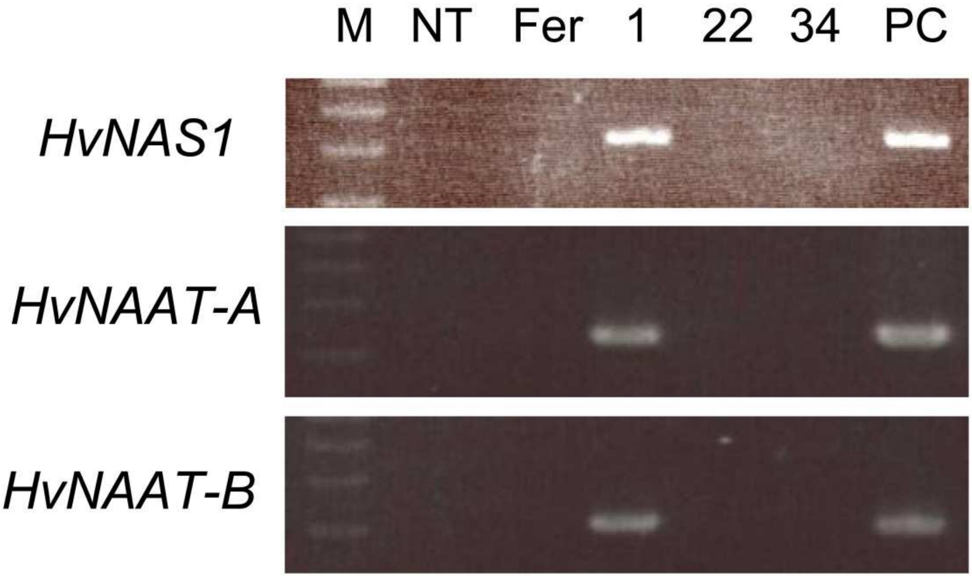

Supplementary Figure 6 | Confirmation of gene insertion by genomic PCR.

M, 100-base pair marker; NT, non-transgenic line; Fer, Fer line 13-6; 1, 22, and 34, Fer-NAS-NAAT-IDS3 lines 1-12, 22-4, and 34-11, respectively; PC, the Fer-NAS-NAAT-IDS3 vector (used as a positive control).

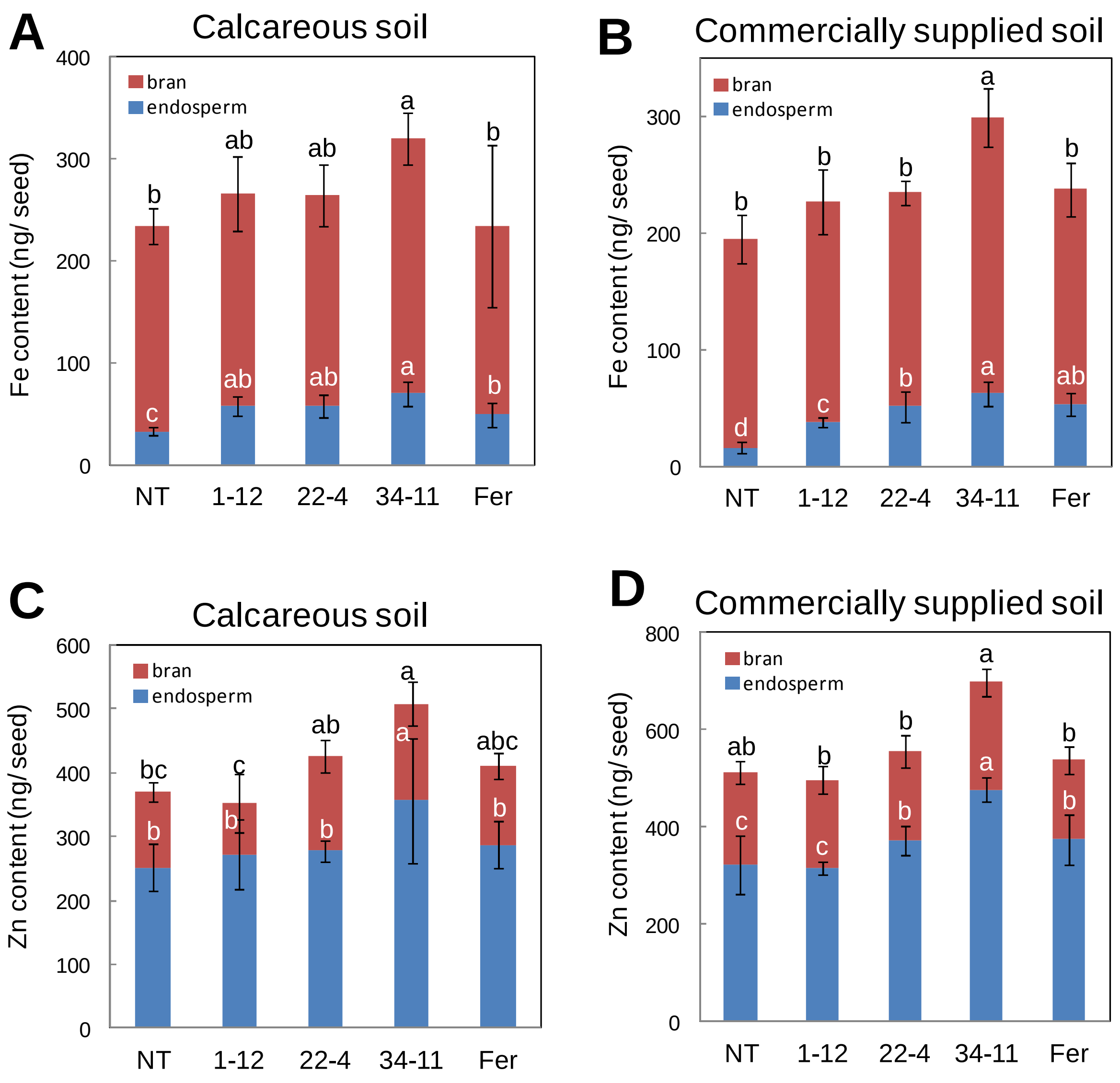

Supplementary Figure 7 | Fe and Zn contents in T_3 seeds.

(A and B) Fe content. **(C and D)** Zn content. Plants were cultivated in calcareous soil **(A and C)** or commercially supplied soil **(B and D)**. Bars represent the means ± standard errors of endosperm or bran metal content from six independent plants (n = 6). NT, non-transgenic seeds; 1-12, 22-4, and 34-11, T_3 seeds of Fer-NAS-NAAT-IDS3 lines; Fer, T_3 seeds of Fer line 13-6. Different white or black letters above the bars indicate significant differences ($P < 0.05$) in endosperm or bran content by Student`s *t*-test for each line.

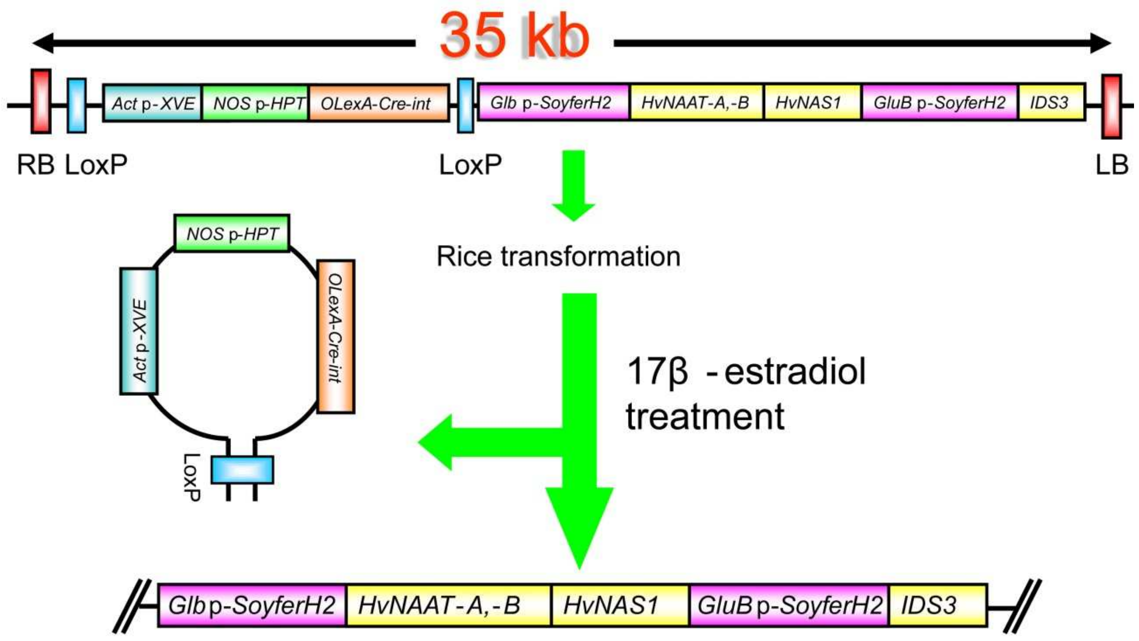

Supplementary Figure 8 | Marker-free vector system.

The names of the genes are described in the legend to **Figure 1**. The T-DNA region between the two *LoxP* sites, which includes *Act p-XVE*, *NOS p-HPT*, and *OLexA-Cre-int*, can be removed by 17β-estradiol treatment (Zuo et al., 2001; Nishizawa et al., 2006). Transgenic rice in which this construct was introduced constitutively expressed the estradiol receptor-based transcription factor XVE under the control of the *OsActin1* promoter. Upon estradiol treatment, activated XVE binds to the *OLexA* promoter region and induces the expression of Cre recombinase, which excises the vector at the two *loxP* regions to form a loop. As a result, the T-DNA region between the two *LoxP* sites, which includes the selection marker gene *HPT*, is removed (Cre/loxP system; Zuo et al., 2001).

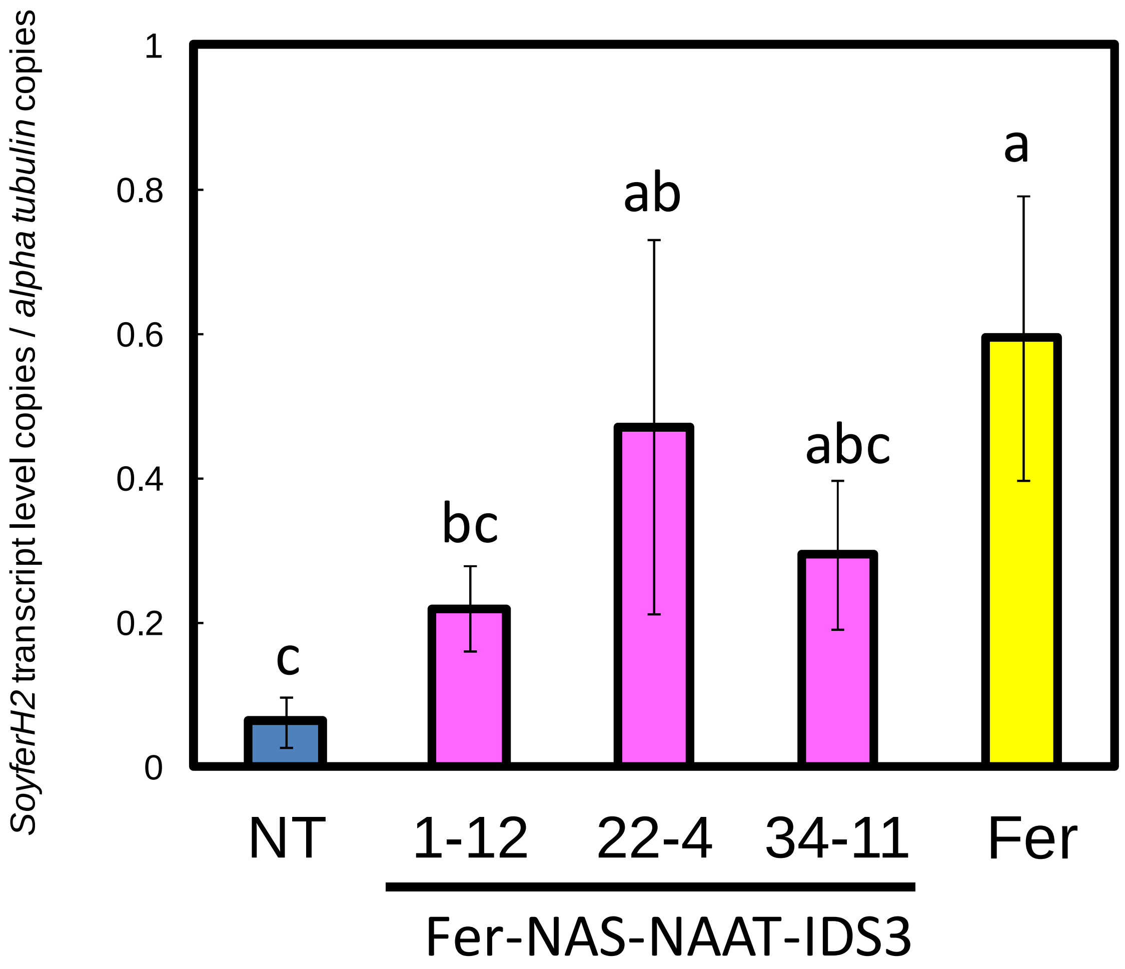

Supplementary Figure 9 | Quantitative real-time RT-PCR analysis of *SoyferH2* in Fe deficient leaves.

T_2 plants were cultivated for 13 days in nutrient solution without Fe. *SoyferH2* expression in leaves was analyzed by quantitative real-time RT-PCR analysis. Bars represent the means ± standard errors of three independent plants for each line. Expression in NT might be attributed to *OsFerritin*. NT, non-transgenic line; 1-12, 22-4, and 34-11, Fer-NAS-NAAT-IDS3 lines; Fer, Fer line 13-6. Different letters above the bars indicate significant differences ($P < 0.05$) by Student`s *t*-test for each line.
